# The Current and Future Status of the Concealed Information Test for Field Use

**DOI:** 10.3389/fpsyg.2012.00532

**Published:** 2012-11-27

**Authors:** Izumi Matsuda, Hiroshi Nittono, John J. B. Allen

**Affiliations:** ^1^National Research Institute of Police ScienceChiba, Japan; ^2^Graduate School of Integrated Arts and Sciences, Hiroshima UniversityHigashi-Hiroshima, Japan; ^3^Department of Psychology, University of ArizonaTucson, AZ, USA

**Keywords:** concealed information test, field application, probative force, statistical judgment, combination of measures

## Abstract

The Concealed Information Test (CIT) is a psychophysiological technique for examining whether a person has knowledge of crime-relevant information. Many laboratory studies have shown that the CIT has good scientific validity. However, the CIT has seldom been used for actual criminal investigations. One successful exception is its use by the Japanese police. In Japan, the CIT has been widely used for criminal investigations, although its probative force in court is not strong. In this paper, we first review the current use of the field CIT in Japan. Then, we discuss two possible approaches to increase its probative force: sophisticated statistical judgment methods and combining new psychophysiological measures with classic autonomic measures. On the basis of these considerations, we propose several suggestions for future practice and research involving the field CIT.

## Overview

The Concealed Information Test (CIT) assesses an examinee’s crime-relevant memory on the basis of differences in physiological responses between crime-relevant and crime-irrelevant items (Lykken, 1959). Although many studies have supported the validity of the CIT, it has not been widely used in field situations. There appear two reasons for its unpopularity. First, some examiners appear to prefer an alternative method termed the Control Question Test (CQT), even though the validity of the CQT has been seriously questioned (Ben-Shakhar, 2002). Second, the CIT is believed to be difficult to apply in non-laboratory field settings. In Japan, however, the autonomic-based CIT is routinely applied successfully in criminal investigations. Even so, CIT results have not been widely influential in court settings.

In this paper, we review the current status of the CIT in the field and laboratory studies, with the goal of outlining steps that can contribute to an increased probative value of the CIT in court. First, we review how Japanese examiners have tried to overcome the difficulties of the CIT for field application. Second, we review statistical methods that can be used to support judgments in field CIT applications, and investigate new measures that can be added to the current CIT implementations.

Throughout this paper, we will emphasize viewpoints relevant to field applications. In the field, an examinee is often not willing to take the test and does not comply with instructions. Therefore, in Japan, a classic autonomic-based CIT has been used, which simply consists of one crime-relevant item and several crime-irrelevant items and does not require an overt behavioral response. This paper will focus on how this existing field CIT can be expanded, but it will not review other alternative approaches. For example, other memory detection or lie detection tests that are still in the laboratory stage, such as the autobiographic implicit association test (Sartori et al., 2008), show promise but are outside of the scope of this paper.

## Current Status of Field CIT

### What is the CIT?

The CIT, also known as the guilty knowledge test (GKT; Lykken, 1959), is used in criminal investigations to examine whether a person recognizes crime-relevant information that innocent people would not know. In the CIT, an examiner presents several items to an examinee, one of which is a crime-relevant item. The items are selected such that innocent examinees would not be able to distinguish the crime-relevant (critical) item from the crime-irrelevant (non-critical) items. Each item is presented once in a block and this block is repeated several times in different presentation orders. During the CIT, the examiner records physiological responses to the items. In the case that the responses do not differ between the critical and non-critical items, the examiner would infer that the examinee does not recognize the critical item. On the other hand, in the case that the responses differ between the critical and non-critical items, the examiner would infer that the examinee recognizes the critical item. Thus, the CIT can provide important forensic information for the police and the justice system, identifying individuals with key information about the crime. Such individuals may be guilty of committing the crime, or have other useful information about the crime if they were not the perpetrator.

The CIT is considered to have a solid scientific foundation, as many laboratory studies have demonstrated its effectiveness (for a review, see Ben-Shakhar and Elaad, 2003). Although published field data are relatively scarce (Elaad, 1990; Elaad et al., 1992; Hira and Furumitsu, 2002; Osugi, 2010), the response pattern of the various physiological measures in field CITs are similar to those observed in laboratory CITs (i.e., skin conductance increase, heart rate decrease, respiration suppression, and finger pulse volume decrease for critical items as compared to non-critical items; Elaad, 1990; Elaad et al., 1992; Osugi, 2010; Verschuere et al., 2011).

### Potential problems in the field application of the CIT

To date, the CIT has not been widely used in field settings. This may reflect, in part, the belief that the CIT is difficult to apply in field settings for a variety of reasons (Krapohl, 2011). First, the CIT can produce false positive cases. Critical items that only a guilty person knows are sometimes difficult to find. Some innocent examinees may know the details of the crime through any number of means, including media reports and rumors (i.e., informed innocent examinees; for a review, see Bradley et al., 2011). Other innocent examinees may, via repeated interrogations or repetitions of crime details, come to have false recollections for crime-relevant items (Allen and Mertens, 2008). If these innocent examinees take the CIT, they would show different responses for critical and non-critical items, resulting in false positive outcomes. Second, the CIT is vulnerable to false negative outcomes. If critical items are selected that are not memorable to the perpetrator of the crime, it is unlikely to be recognized, thus producing a false negative outcome. Even if examinees do have crime-relevant memories and recognize the crime-relevant item, physiological differences sometimes might not be observed. For example, although skin conductance is typically measured in the CIT, one study reported that approximately one out of four people were electrodermal non-responders to orienting stimuli (Venables and Mitchell, 1996). Third, some studies have shown that the CIT is vulnerable to physical countermeasures (e.g., pressing the toes against the floor when non-critical items are presented) as well as mental countermeasures (e.g., counting numbers each time a non-critical item appears; for a review, see Ben-Shakhar, 2011). In the next section, we will introduce how Japanese CIT examiners have attempted to overcome these three problems.

### Current field use of the CIT in Japan

In spite of the three problems outlined above, the CIT has been officially and systematically used in Japan for the last 50 years. About 100 trained examiners perform about 5,000 CITs per year (Osugi, 2011). All examiners (who are not investigators) belong to a forensic science laboratory of a prefectural police headquarter. The CQT (Reid, 1947) is no longer used. The results of the CIT have been accepted as evidence in court since the 1960s. Although Japan’s successful application of the CIT in the field has attracted attention from foreign researchers and examiners, not much has been written about how the potential problems for field use of the CIT have been addressed in Japan. Therefore, potential solutions are reviewed briefly below, and more details are available from Osugi (2011).

#### Prevention of false positive cases

Japanese CIT examiners make every effort to prevent false positive cases through every step in the process, from pre-exam preparation to the actual administration of the CIT. On a routine basis, an examiner advises criminal investigators to conduct the CIT at an early stage of the investigation in order to make it less likely that crime-relevant items become known to a wider audience over time. When an examiner is requested to conduct the CIT, he/she first consults with investigators. An examiner also checks media reports related to the crime and to the record of investigation. Furthermore, before conducting each CIT, an examiner presents all the items in the CIT to an examinee, and asks the examinee if there are items that he/she recognizes or feels different from the others. If the examinee points out the crime-relevant item, the examiner would not administer the CIT question about that item.

#### Prevention of false negative cases

Japanese CIT examiners strive to select critical items that a guilty person should remember. They try to avoid using peripheral features of the crime, and instead use central features as critical items (Carmel et al., 2003; Gamer et al., 2010; Nahari and Ben-Shakhar, 2011). In addition, before each CIT, an examiner explains the meaning of each question item to an examinee, in order that the examinee will understand what the examiners are asking.

However, even when an examinee might recognize a critical item, he/she sometimes may not show a different physiological response between the critical and non-critical items. One of the strategies to avoid this type of false negative case is the simultaneous measurement of multiple validated responses. In Japan, a new polygraph system has been used since 2003, which simultaneously records skin conductance, heart rate, pulse volume, and respiration. These measures are thought to reflect the different aspects of a physiological response. Laboratory studies show that combining these multiple measures could reduce false negative rates while maintaining low false positive rates (e.g., Gamer et al., 2008a).

#### Counter-countermeasures

To guard against physical countermeasures, an examiner monitors an examinee’s behavior and his/her physiological responses carefully during the CIT. When the examiner thinks that the examinee is intentionally applying countermeasures (e.g., frequent body movements, sighs, or sniffing), he or she would instruct the examinee to refrain from such activities (Osugi, 2011). Although specific sensors to detect physical countermeasures have not been applied in Japan yet, it may be useful to introduce, for example, pressure-based sensors incorporated in the test chair and floor pads, which have been used in some other countries.

Previous studies have suggested that mental countermeasures affect skin conductance, but do not affect respiration (Ben-Shakhar and Dolev, 1996; Honts et al., 1996). In Japan, an examiner measures multiple autonomic indices including respiration, which can serve to lessen the chance that countermeasures will change the outcome of the CIT. To measure an examinee’s physiological response from various response channels can thus contribute to reducing the effect of unobservable mental countermeasures.

#### Other attempts

Examiners in Japan also use other procedures to get more accurate and/or informative results. First, examiners always conduct a pretest before asking about crime-relevant information. In the pretest, an examiner asks an examinee to memorize a number on a card in private and then presents several numbers including the memorized number. The pretest not only helps the examinee to understand the CIT paradigm, but also helps the examiner to know the physiological response pattern of the examinee when he or she recognizes an item. Considering the response pattern, the examiner conducts the subsequent CITs. For example, if the examinee showed high reactivity in skin conductance response in the pretest, the examiner judges the responses of subsequent CITs paying more attention to the skin conductance response.

Second, an examiner sometimes uses a *searching CIT*. The searching CIT is different from the typical CIT in that an examiner does not know which item is crime-relevant in advance. For example, if a weapon has been missing, an examiner can ask an examinee about the place where he/she abandoned a weapon, such as “Was a weapon abandoned in area A, area B, …, or area E?” Indeed, the judgment is more difficult for a searching CIT than for a usual CIT with known solutions, because the examiner has to judge not only whether the examinee has recognition but also which item the examinee recognizes. Additionally, in the case that the question items do not cover all possibilities, the finding of no physiological differences between items cannot support an examiner’s conclusion “the examinee does not recognize the crime-relevant item;” instead, this finding can only support the conclusion that “the examinee does not recognize any items in this question set.” But if an examiner develops an appropriate question set, the searching CIT can suggest potential new crime-relevant information of which even investigators have no knowledge. In the above example, if the responses differ between area A and other areas, the investigators will focus investigation on area A and consequently may find the missing weapon.

Third, in Japan, an examiner only decides on whether an examinee recognizes each crime-relevant item and never integrates the results of multiple CIT questions to judge whether the examinee is guilty or innocent. It is the investigators’ task, rather than the examiner’s task, to integrate the results across the CIT questions and evaluate the examinee’s likelihood of guilt. Some authors, however, have argued that examiners should integrate results across multiple CIT questions in order to obtain more statistically reliable and robust results (Ben-Shakhar and Elaad, 2002). However, Japanese examiners have maintained the approach of only adopting a judgment for each CIT question. One of the justifications for conducting the test in this manner is that it allows the examiner to clarify which items the examinee recognizes and which items the examinee does not. For example, in the case of a theft that was conducted by a group of perpetrators, information indicating whether the examinee knows each crime-relevant item may become a clue to reveal what role he/she played in the crime (e.g., a major culprit or just a lookout). Thus, treating results from each CIT question separately can facilitate investigations of cases involving multiple suspects, and provide details to guide and facilitate the investigators’ continuing inquiries for any type of case. Additionally, as described above, Japanese examiners sometimes use searching CITs; in such cases where an examiner does not know with certainty which alterative is the critical item for a given CIT question, it is difficult to integrate CIT results across questions.

#### Validity of the field CIT in Japan

One article has reported on field CIT datasets using the current polygraph system in Japan. Kobayashi et al. (2009) analyzed the data of 113 CIT questions obtained from 38 examinees (33 men and 5 women, mean age = 36.4, *SD* = 12.5). Subsequent investigations confirmed that all of these examinees recognized the critical items of these CIT questions. For each CIT question, the responses were compared between critical and non-critical items with a *t* test. If the *p* value did not exceed 0.10, the examinee was judged as recognizing the critical item. The correct detection rates were 52.5% for the skin conductance response, 49.5% for heart rate (average in 16–20 s after the item onset), 38.1% for respiration line length (average in 0–15 s), and 26.2% for normalized pulse volume (average in 6–10 s). It should be noted that these values are correct detection rates (i.e., sensitivities) for individual CIT questions using a single measure. Although Kobayashi et al. did not report the data, combining the various physiological measures should increase the overall detection rate. In the actual field CIT, examiners arrive at a conclusion by combining all of the available measures. In addition, to address the specificity of the CIT (i.e., how well each measure correctly indicates non-recognition of critical items when examinees do not have recognition), a larger dataset including both guilty and innocent subjects would be required.

## Improving the Probative Force of the CIT in Court

Although the CIT has been widely used for criminal investigations and its results have been sometimes accepted as evidence in court in Japan, the CIT results are not considered sufficiently strong that they typically directly affect the outcomes in court. To improve the probative force of the CIT, we believe the following two approaches are most promising.

The first approach is to use statistical methods to interpret the results. In field use of the CIT in Japan, CIT results are mainly derived through the examiners’ visual inspections (Osugi, 2011). If the judgment is underpinned by statistical methods, the CIT results would become more convincing for judges. Moreover, such an approach is well-justified in the literature: statistical actuarial judgment has greater reliability and validity than judgments based on visual impressions (Dawes, 1979). In laboratory studies, Lykken’s scoring and *z-*score averaging have been commonly used for decision-making (Meijer et al., 2011). Lykken, 1959 scoring is based on the rank of the critical item among all items in descending order of the response values. *Z-*score averaging uses the average standardized response value across blocks and measures (Ben-Shakhar, 1985). Although these two methods are simple and clear, they do have drawbacks. We will review these two methods critically and compare them with other proposed methods below.

The second approach is to add new measures to current field CIT to increase its accuracy. In the current field CIT, heart rate, skin conductance, respiration, and pulse volume are recorded. New measures can be introduced either by improving quantification methods of currently recorded responses or by recording new response channels, such as reaction time, facial responses, activations using functional magnetic resonance imaging (fMRI), and features of the electroencephalogram (EEG) and event-related potential (ERP). We will review these new measures and evaluate these from the viewpoint of field application.

### Statistical evaluation methods

Here, we review statistical methods that have been used in previous studies. First, we review standard statistical methods such as Lykken’s scoring and *z-*score averaging. We then review five other proposed methods: logistic regression discrimination, latent class discrimination, Bayesian classification, multivariate normal distribution discrimination, and dynamic mixture distribution discrimination. Finally, we outline recommendations for their use.

#### Standard statistical methods

##### Lykken’s scoring method

**Figure 1:**
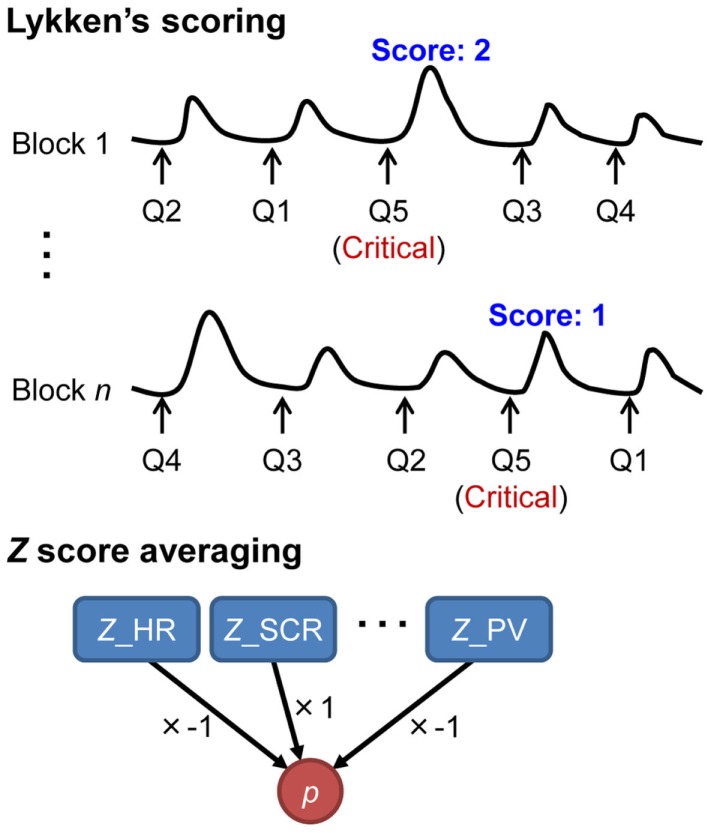
**Illustrations of the standard statistical methods: Lykken’s scoring and *z-*score averaging**. *Z*_HR, a *z*-score for heart rate; *Z*_SCR, a *z*-score for skin conductance response; *Z*_PV, a *z*-score for pulse volume; *p*, probability. Lykken’s scoring assigns a score of 2 if the critical item elicited the largest response, a score of 1 if the critical item elicited the second largest response, and a score of 0 otherwise in each block. In *z-*score averaging, *z-*scores are simply averaged across blocks and measures. *Z*-scores may be multiplied by −1 if a smaller response is characteristic of recognition.

This is a traditional discrimination method proposed by Lykken (1959; Figure 1). This method assigns a score of 2 if the critical item elicited the largest response, a score of 1 if the critical item elicited the second largest response, and a score of 0 otherwise in each block. If the average of the scores across blocks exceeds a threshold, it is judged that the examinee recognizes the critical item.

Lykken’s scoring method has several advantages. First, this method is very practical. It can be used without quantification and parameter estimations. Second, because responses are ranked within each block, correction is not required even if physiological levels change between blocks as a result of habituation.

However, Lykken’s scoring method has its drawback: this method does not take into account quantitative differences between responses to critical and non-critical items (Meijer et al., 2011). For example, even when the response to the critical item might be three times as large as the next largest response, the score would be the same as when it is only slightly larger.

##### *Z*-score averaging

*Z-*score averaging is widely used in laboratory studies to capture quantitative differences between items (Ben-Shakhar, 1985; Figure 1). In this method, a response to each item is first standardized using the mean and SD of each measure within a block. The aim of the standardization is (1) to cancel out the differences in physiological levels among blocks and (2) to treat multiple measures that have different units in the same dimension. If a measure typically decreases to a critical item (e.g., heart rate, respiration, or pulse volume), its *z-*score is multiplied by −1. The scores for the critical item are then averaged across all blocks and all measures. We then judge whether the averaged *z-*score is significantly high enough to exceed typical cut points using the standard normal distribution. This method needs no parameter estimation *a priori* and thus is easy to apply to field CIT.

However, this method has two disadvantages. First, this method assumes that for every subject, all measures respond in the normative expected direction. It thus does not consider individual differences in response patterns. The physiological measures that respond distinctively between critical and non-critical items are sometimes different between examinees (Matsuda et al., 2006). For example, Osugi (2011) reported results from field data in which a guilty examinee showed constant distinctive responses only in respiration. In such a case, with an increasing number of measures, the average *z-*score will become smaller and thus might lead to a false negative. Second, this method does not consider the differences in general accuracies among measures. For example, in laboratory studies, accuracy is usually higher for skin conductance than for other measures (i.e., heart rate, respiration, and pulse volume; e.g., Ben-Shakhar and Elaad, 2003; Gamer et al., 2008b). However, with *z*-score averaging, all measures are weighted equally. It might be preferable if each measure were weighted according to its accuracy.

#### Proposed statistical methods

**Figure 2:**
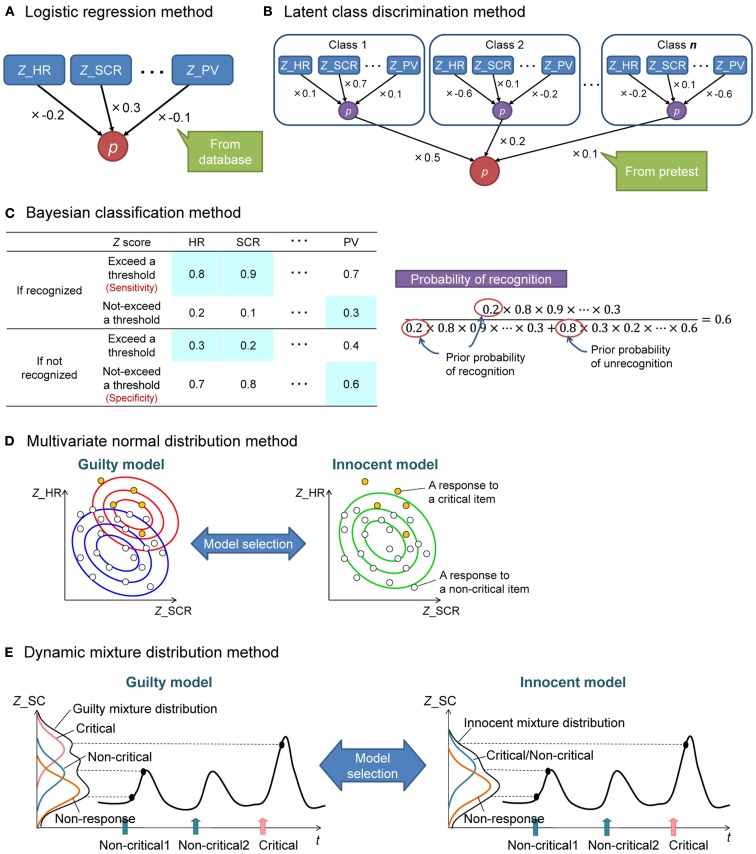
**Illustrations of the five proposed statistical discrimination methods**. *Z*_HR, a *z*-score of heart rate; *Z*_SCR, a *z*-score for skin conductance response; *Z*_PV, a *z*-score for pulse volume; *p*, probability. **(A)** The logistic regression method is similar to *z-*score averaging, but each *z-*score is weighted according to the accuracy of the measure estimated from previous datasets. **(B)** The latent class discrimination method is a two-layer model of the logistic regression method. There is an appropriate regression formula for each class, and the result of the regression formula is summed across classes with a weight of the likelihood of an examinee belonging to a class according to his/her pretest result. **(C)** The Bayesian classification method calculates the probability of recognition by multiplying prior probabilities and the probabilities that a standardized response value of each measure exceeds/does not exceed a threshold in the recognition condition. Here is the case that a participant’s heart rate change and skin conductance response exceeded the threshold, while his/her pulse volume did not exceed the threshold. **(D)** In the multivariate normal distribution method, a guilty model (two-distribution model) and an innocent model (one-distribution model) are applied to the obtained responses in a CIT (each small circle represents a response to a critical (yellow) or a non-critical (white) item). The better fitted model will be selected. **(E)** The dynamic mixture distribution method uses time series and is an extended version of the multivariate normal distribution method. In this method, a guilty model (representing time series with a mixture of three distributions) and an innocent model (representing time series with a mixture of two distributions) are applied to the obtained time series in a CIT. The model that fits the time series best is selected.

To overcome the disadvantages of *z-*score averaging, other statistical methods have been proposed: logistic regression discrimination, latent class discrimination, Bayesian classification, multivariate normal distribution discrimination, and dynamic mixture distribution discrimination. We will explain these methods below and in Figure 2, and evaluate these methods from the viewpoint of field application. In particular, we will focus on whether a new method overcomes the limitations of *z-*score averaging.

##### Logistic regression discrimination

This method considers the differences in accuracy among measures by allocating a weight to the *z-*score of each measure (Gamer et al., 2006, 2008b; Figure 2A). The weights are acquired from the CIT datasets of previous examinees, where ground truth has already been established. Each weight reflects the effectiveness of the measure for estimating recognition. If these weights are all 1, the result will be the same as the one of *z-*score averaging.

This method is practical and widely used in various research domains. If the sample size is large, the weight parameters will be estimated quite stably.

On the other hand, this method does not sufficiently consider individual differences in response patterns. This is because the parameters are calculated to be fitted to the normative response pattern. Similar to *z-*score averaging, if a guilty examinee shows distinctive responses only in a small number of measures, this method might produce a false negative. Additionally, the logistic regression method may underperform the *z*-score averaging if the sample size is not large enough to reliably estimate the parameters (c.f., Dawes, 1979).

##### Latent class discrimination

This method is an extended version of the logistic regression model that considers individual differences in response patterns. As mentioned before, in the field CIT, an examiner conducts a pretest using cards to capture the response pattern of an examinee. However, the results of the pretest are not considered in most statistical methods. Therefore, Matsuda et al. (2006) proposed the latent class discrimination method (Figure 2B). In this method, previously obtained examinees are grouped into several classes, for each of which a discriminant formula (e.g., logistic regression formula) is calculated and fit to the response pattern of the examinees belonging to that class. It is then estimated if a given examinee recognizes a critical item using the following process. First, the probability that the examinee would recognize the critical item is computed by applying the discriminant formula of each class to his/her standardized response values. Second, the probability that the examinee belongs to a class is computed by using his/her pretest data. Finally, the recognition probability is calculated by summarizing each class’s recognition probability across all classes with a weight of the probability for the class that the examinee belongs to. In this manner, each examinee can be distinguished through his/her response pattern.

This method considers several response patterns as latent classes. In addition, the accuracies of the measures have been reflected as parameters of a discriminant formula in each class. Moreover, these parameters can be estimated stably with a large dataset of previous examinees.

However, factoring in the pretest data can also become a drawback in practical applications. In Japan, about 5–6 CITs are typically conducted after the pretest. It takes about 2 or 3 h to finish all the CITs (Osugi, 2011). Therefore, a response pattern may change from the pretest to the last CIT for an examinee. In addition, this method is based on a more complex, hierarchical model, and consequently needs to estimate more parameters than the logistic regression method. This implies that the latent class discrimination method requires a larger dataset than the logistic regression method for parameter estimation.

##### Bayesian classification

This method combines multiple measures by using computations based on Bayes’ theorem (Allen et al., 1992; Figure 2C). This approach calculates the probability that an examinee recognizes an item using (1) the sensitivity/specificity of each measure (i.e., the probability that a response value exceeds (or does not exceed) a threshold in the condition that an examinee recognizes (or does not recognize) the item) and (2) a prior probability (i.e., the probability that the examinee shows the distinctive response by chance to each item, which is determined by the number of items in the test). This method also uses a within-subjects standardization, so that large individual differences in response magnitude are eliminated, and the pattern of responses across critical and non-critical items is retained. First, for each standardized measure, the sensitivity, specificity, and threshold are calculated from a previously obtained dataset. The standardized response value of a given examinee is then compared to the threshold. If the response value exceeds (or does not exceed) the threshold, the sensitivity (or 1−sensitivity) is entered into Bayes’ formula to calculate recognition probability. Similarly, the specificity or 1−specificity can be entered into Bayes’ formula to calculate the probability of a failure to recognize crime-relevant items.

As this method treats responses as binary data – that is, whether a response exceeds the threshold or not – quantitative differences between items are not fully captured with this method. On the other hand, thanks to dealing with binary values, this method is not excessively affected by outliers. Controlling the influence of factors that will produce outliers is difficult in the field situation as compared with the laboratory situation. For this reason, for field CIT applications, the Bayesian classification may be preferred to the other statistical methods.

##### Multivariate normal distribution discrimination

In contrast to logistic regression, latent class discrimination, and Bayesian classification, which require previously obtained data to estimate their parameters, the multivariate normal distribution method requires only the CIT results of the current examinee (Adachi, 1995; Figure 2D). If the examinee recognizes a critical item, the distribution of the responses should differ between critical and non-critical items (i.e., *guilty model*). In contrast, if the examinee does not recognize the critical item, the distribution should not differ between critical and non-critical items (i.e., *innocent model*). Both the guilty model and the innocent model are applied to the given responses in the CIT. If the guilty model better fits the responses than the innocent model, the examinee is judged as recognizing the critical item.

This method only requires that responses to critical and non-critical items differ, and does not require a previous dataset. In addition, this method has no assumptions of typical response patterns. Therefore, it can deal with various response patterns, even if the response pattern is very different from the typical normative pattern.

However, with this method, we can estimate model parameters (i.e., mean and SD of distributions) only from the given data. The sample size is thus the number of repetitions; for example, if each item is repeated five times, the sample size is five, which is too small to be used to estimate stable parameters. In addition, although the accuracy of each measure can be calculated based on previous datasets, this method does not use previous datasets. Therefore, the differences in accuracy between measures cannot be taken into account.

##### Dynamic mixture distribution discrimination

In order to estimate stable model parameters by using only the given data, the extended version of the multivariate normal distribution method – the dynamic mixture distribution method – was developed (Matsuda et al., 2009a; Figure 2E). Similar to the multivariate normal distribution method, this method prepares a guilty model and an innocent model, but applies these models to time series data. The guilty model represents the response time series using three distributions: a non-response distribution corresponding to the base level, a critical response distribution corresponding to responses to the critical item, and a non-critical response distribution corresponding to responses to the non-critical items. In contrast, the innocent model represents the response time series using two distributions: a non-response distribution and a pooled critical/non-critical response distribution corresponding to responses to both critical and non-critical items. The guilty and innocent models are applied to the time series of the CIT data. If the time series is more compatible with the guilty model than with the innocent model, the examinee is judged as recognizing the critical item.

Similar to the multivariate normal distribution, this method requires no previous dataset and no assumption of typical response patterns. Therefore, this method is very flexible and can easily accommodate individual differences in response patterns, even if an individual’s response pattern is very different from the typical normative response pattern. Additionally, because time series data are used, stable model parameters may be estimated with the typical number or repetitions in the CIT.

However, since this method does not depend on previous datasets, the accuracy of each measure cannot be taken into account. Furthermore, this method requires complex calculations for parameter estimations (i.e., Gibbs sampler). Given current technology, it takes at least about 10 min to finish the calculation of the parameters. If the calculation algorithm is improved, this method might be ideally suited to field CIT use.

#### Summary of statistical methods

Table 1 summarizes the advantages and disadvantages of the various statistical methods. As the table shows, a perfect statistical method does not exist. More studies are required to continue to improve existing methods.

**Table 1:** **Comparison of statistical methods in terms of features that are important for field application**.

Statistical method	Flexibility for individual differences	Consideration of accuracy differences among measures	Need of previous dataset for parameter estimation	Stability of parameter estimation	Complexity of model
Standard: *Z-*score averaging	Low	No	No	No parameters	Low
(A) Logistic regression	Low	Yes	Yes	Stable	Medium
(B) Latent class discrimination	High (assume subgroups having different response patterns)	Yes	Yes	Stable	High
(C) Bayesian classification	Medium	Yes	Yes	Stable	Medium
(D) Multivariate normal distribution	High (no assumption of a typical response pattern)	No	No	Unstable	Medium
(E) Dynamic mixture distribution	High (no assumption of a typical response pattern)	No	No	Stable	High

However, the most promising method at present would appear to be the latent class discrimination method or the dynamic mixture discrimination method. Table 1 shows the methodological advantages of the latent class and dynamic mixture distribution methods as compared to the other methods, recognizing that their parameter calculations are complex. Furthermore, superiority of these two methods in terms of discrimination performance was demonstrated empirically (Matsuda et al., 2009a). In this study, 19 guilty participants were discriminated from 15 innocent participants by using the logistic regression, latent class, multivariate normal distribution, and dynamic mixture distribution methods. The discrimination performance was higher for the latent class and for the dynamic mixture distribution methods than for the logistic regression and the multivariate normal distribution methods. Of course, this result should be verified by using larger number of field CIT datasets. In addition, their discrimination performance should be also compared with that of the Bayesian classification method, which is expected to be robust in the face of outliers.

Methods requiring previously obtained datasets may have limited utility for filed CIT applications. Such methods (i.e., the logistic regression, latent class, and Bayesian discrimination methods) require the parameters to be estimated from the field CIT data for which valid ground truth data are available for each examinee. However, the exact confirmation of this knowledge is very difficult to obtain in the field situation, since it is difficult to know with absolute certainty who is guilty and who is innocent in a field case. It may take a rather long time to collect a sufficient number of appropriate field datasets for parameter estimation. If the parameters are estimated from an insufficient number of field samples, these methods may underperform the simple *z-*score averaging (Dawes, 1979). In contrast, methods that require only the current dataset (i.e., the multivariate normal distribution and dynamic mixture distribution method) have a strong advantage for field use since they do not require a previously obtained dataset. But this also indicates that the latter methods may be more influenced by missing values and measurement artifacts than the former methods. Even when adopting the latter methods, evaluating their generalizability will require using a field dataset.

### Additional measures

In order to improve the probative force of the CIT in court, it would be also promising to use additional measures that can potentially increase the accuracy of the CIT. The current field CIT, that is based on measures of autonomic responses (i.e., skin conductance, heart rate, respiration, pulse volume), has been working well so far in Japan. Therefore, it would be more promising to add new measures to the autonomic-based CIT instead of altering the current field CIT completely to use alternative measures. In this section, we will review additional CIT measures that can be obtained by using two approaches. The first approach is to refine the quantification of the classic autonomic responses. The second approach is to implement new physiological measures to augment the autonomic responses used currently.

#### Quantification of new/refining aspects of autonomic responses

The Improvement of current quantification methods is a simple way to increase accuracy of the current test. Here, we will review some examples of how quantification might be refined.

##### Respiration

Respiration has been operationalized as respiration line length in almost all CIT studies (for a review, see Gamer, 2011a). The respiration line length is defined as the sum of the moving distances of the respiration curve in a specified time interval. The respiration line length decreases when respiration is suppressed (i.e., shorter respiratory time and smaller amplitude), and thus is a good measure for the CIT. However, the line length is biased by how the parts of the respiratory cycles are included in the time interval. To account for this bias, Elaad et al. (1992) shifted the starting point of the time interval slightly, calculated the line length for each shift, and then averaged the line lengths for all shifts. However, even this method cannot remove the bias completely (Figure 2 in Matsuda and Ogawa, 2011).

To fully resolve this bias problem, a new quantification method – a weighted average respiration line length – has been recently proposed (Matsuda and Ogawa, 2011). This method calculates the respiration line length per cycle, weights it with the proportion that the cycle occupies in the time interval, and then averages the weighted line lengths across all cycles involved in the time interval. The discrimination performance was significantly better for the weighted average respiration line length than for the traditional respiration line length.

Moreover, there is an undeniable possibility that changes in respiratory rate and amplitude are elicited independently in the CIT. To extract more precise information from respiration, respiratory rate, and amplitude could be measured separately. In order to quantify these, the use of the weighted average method would be preferable (e.g., Matsuda et al., 2009a).

##### Pulse volume

Recently, pulse volume has been quantified as finger pulse waveform length in a way similar to that of respiration line length (Elaad and Ben-Shakhar, 2006; Vandenbosch et al., 2009). The finger pulse waveform length can reflect both pulse rate and amplitude information. As mentioned above, the line length is affected by which proportion of a cycle is included. However, the effect of this bias is much smaller for pulse volume than for respiration, because the cycle time of a pulse is much shorter. On the other hand, since heart rate is computed with an electrocardiogram in Japan, the measurement of finger pulse volume length is redundant.

In Japan, normalized pulse volume has been applied to the field CIT to evaluate vascular tone more accurately. The normalized pulse volume is computed per pulse cycle by dividing the amplitude of the cycle by the average voltage during the cycle. The normalized pulse volume is advocated as a more valid measure for the assessment of vascular tone than the usual pulse volume (Sawada et al., 2001). The validity of the normalized pulse volume has also been confirmed in a CIT study (Matsuda et al., 2009a).

#### Adding new measures

New physiological or behavioral measures can be recorded in addition to autonomic responses in the field, particularly if the recording is easy and stable. Here, we will review reaction time, facial features, fMRI activations, and EEG/ERP features.

##### Reaction time

One possible measure that has been considered is reaction time after item onset (for a review, see Verschuere and De Houwer, 2011). Some studies reported high accuracy of individual classification using reaction time. For example, Allen et al. (1992) reported a sensitivity of 0.950 and the specificity of 1.000.

However, in the current situation in the field, there may be problems with using reaction time. First, reaction time can be controlled intentionally. It might therefore be easier to use countermeasures that affect reaction time than those that affect autonomic responses. In fact, some studies use the response time as a measure of countermeasures (Rosenfeld et al., 2008; Winograd and Rosenfeld, 2011). Second, it is uncertain whether examinees would follow the instructions, such as “respond as quickly and accurately as possible.” Unlike the autonomic-based CIT, a reaction-time task requires examinees to respond actively. Even when examinees are innocent, however, they may not take the test willingly and thus may not cooperate. In addition, attributes of field examinees are more diverse than those of participants in laboratory studies. For example, elder examinees have slower and more variable reaction-times, which might render this measure less useful in some populations.

Despite these limitations, research might profit from further examination of reaction time in the CIT. It is an easily obtained measure, and individual differences in response times might not be of concern if quantified using within-subject metrics (*z*-scores). Moreover, it might be possible to identify reaction-time response patterns that would suggest when reaction time can, and when it cannot, provide useful information.

##### Facial features

Facial expressions have potential as a measure in current field CIT examinations. Because a face is usually not covered, it is easy to record the information without attaching special electrodes (i.e., with a remote-sensing technique).

It is well-known that lie detection can make use of facial muscle activity (Ekman, 2001). However, as far as we know, no study has reported the use of facial muscle changes in the CIT, but automated Facial action coding system (FACS; Littlewort et al., 2011) might make this an easy possibility to explore further. On the other hand, facial skin surface temperature has been measured in the CIT (Pollina et al., 2006). In this study, the temperature increased for critical items compared to non-critical items in a region below the eyes. Its individual classification result was a sensitivity of 0.917 and a specificity of 0.917.

Information related to the eyes has also been applied to the CIT. Startle eye blinks reduced more for critical items than for non-critical items (Verschuere et al., 2007). Temporal distributions of blinks differed between critical and non-critical items (Fukuda, 2001). Pupil sizes increased more for critical items than for non-critical items (Bradley and Janisse, 1981; Lubow and Fein, 1996). Lubow and Fein (1996) reported a sensitivity of 0.50–0.70 and a specificity of 1.00 using pupil sizes.

Thus a variety of facial measures show some promise for use in the CIT, but none have been extensively researched. Therefore, future research should determine if use of these facial measures can increase the validity of the current autonomic-based CIT.

##### fMRI

Recent research has utilized fMRI in CIT-like experiments (for a review, see Gamer, 2011b). Nose et al. (2009) reported the accuracy of fMRI in the CIT: the sensitivity was 0.84 and the specificity was 0.84. However, the use of fMRI in the field would be difficult at the present time. First, the equipment for fMRI is expensive and not portable. Second, examinees must be extremely cooperative as they are not able to move during the fMRI scanning and would have to tolerate the noise during the test. Third, some examinees could not be tested if they have metal in their bodies that would make fMRI unsafe. Although technical improvement of recordings and analyses are expected in future research, fMRI measures may inherently carry no more or no less weight than other measures used in the CIT.

##### EEG/ERPs

Many laboratory studies have measured EEG during the CIT and reported significant differences in ERP components between critical and non-critical items, especially P3 amplitudes (Rosenfeld et al., 1988; Farwell and Donchin, 1991; Allen et al., 1992; Rosenfeld, 2011). A recent meta-analysis showed that the P3 measure is more effective than the traditional autonomic measures in detecting participants’ concealed knowledge: Cohen’s *d* was 2.55 for the P3 amplitude and 1.72 for skin conductance response (Ben-Shakhar and Meijer, 2012). This result is similar to that of Allen and Iacono (1997), in which they compared the area under ROC curve from their ERP data to published skin conductance data. The increase of the P3 amplitude is thought to reflect the significance of the critical item for the examinees (Rosenfeld, 2011), which is often embedded within an oddball paradigm. In addition, recent studies with rather long inter-stimulus intervals (>7 s) reported the increase of the N2 (Matsuda et al., 2009b, 2012; Gamer and Berti, 2010) and the late positive potential (Matsuda et al., 2009b, 2012) for the critical item.

Due to the progress of recording and analysis techniques it has become easier to measure EEG in field situations. In fact, an EEG can be recorded with a polygraph system currently used in field CIT in Japan, although the stimulus presentation/control system for it has not been equipped yet. A recent study measured ERPs under the standard protocol of the autonomic-based field CIT (Matsuda et al., 2011). This study showed that late positive potential significantly differed between critical and non-critical items, even when each item was presented only five times. Importantly, including the late positive potential improved the discrimination performance of the standard autonomic-based CIT. Furthermore, Rosenfeld (2011) have proposed a new protocol of the ERP-based CIT in order to make the test resistant to countermeasures (“complex trial-based CIT”), and have reported high accuracies. Collectively these studies indicate that features of the ERP would be promising additions to the field CIT.

Moreover, although most studies quantified EEG in the time domain, some recent studies focused on information in the frequency domain (Abootalebi et al., 2006, 2009; Zhao et al., 2011). These studies show that differences in wavelet features can reflect the differences between critical and non-critical items. Furthermore, the frontal asymmetry of left and right EEG alpha power may have promise as a new measure. Frontal EEG asymmetry is an index of the basic emotional dimension of approach versus withdrawal (Coan and Allen, 2004). In the CIT, relative right frontal alpha activity was significantly lower for critical items than for non-critical items (Matsuda et al., submitted). This result suggests that the critical item would elicit withdrawal-oriented motivation and emotion, which may be an additional indicator of recognition of the critical item.

## Summary

In the present paper, we reviewed how the CIT has been used for field criminal investigations in Japan, and suggest that with appropriate training and institutional support, the CIT can frequently be used in field applications. We also reviewed various statistical methods and potential new measures, which may contribute to improved validity and increased probative value of the CIT. We suggested that more studies of these various statistical methods are required before applying the statistical methods in the field. We also highlighted the promise of adding new quantification of existing measures and adding new measures such as EEG/ERP indices to the current field CIT. It should be an immediate goal of the Japanese CIT examiners and researchers to improve the probative value of the field CIT by introducing statistical judgment methods and then adding new measures to the current CIT.

Despite improvements in measures and statistical assessment, it is important to remember that the CIT is not a test to judge whether an examinee is guilty or innocent. The CIT can show only with relatively high probability whether the examinee recognizes the crime-relevant item. The examinee may have obtained crime-relevant information by any number of means, only one of which is by being the perpetrator of the crime, while others include accidental exposure via media or interrogations, or exposure via a relationship with the perpetrator of the crime; a good examiner of course pays close attention to remove these possibilities. However, the CIT result can be used as one scientific indicator of whether an individual may have been involved in the crime under investigation. Given the fundamentally sound paradigm of the CIT, and the promise of improvements using more sophisticated statistics and additional measures, we hope that the use of the CIT will increase, with Japan’s implementation serving as a useful model.

## Conflict of Interest Statement

The authors declare that the research was conducted in the absence of any commercial or financial relationships that could be construed as a potential conflict of interest.

## References

[B1] Abootalebi V. Moradi M. H. Khalilzadeh M. A. (2006). A comparison of methods for ERP assessment in a P300-based GKT. Int. J. Psychophysiol. 62, 309–320 10.1016/j.ijpsycho.2006.05.009 16860894

[B2] Abootalebi V. Moradi M. H. Khalilzadeh M. A. (2009). A new approach for EEG feature extraction in P300-based lie detection. Comput. Methods Programs Biomed. 94, 48–57 10.1016/j.cmpb.2008.10.001 19041154

[B3] Adachi K. (1995). Statistical classification procedures for polygraph tests of guilty knowledge. Behaviormetrika 22, 49–66 10.2333/bhmk.22.49

[B4] Allen J. J. B. Iacono W. G. (1997). A comparison of methods for the analysis of event-related potentials in deception detection. Psychophysiology 34, 234–240 10.1111/j.1469-8986.1997.tb02137.x 9090275

[B5] Allen J. J. B. Iacono W. G. Danielson K. D. (1992). The identification of concealed memories using the event-related potential and implicit behavioral measures: a methodology for prediction in the face of individual differences. Psychophysiology 29, 504–522 10.1111/j.1469-8986.1992.tb02024.x 1410180

[B6] Allen J. J. B. Mertens R. (2008). Limitations to the detection of deception: true and false recollections are poorly distinguished using an event-related potential procedure. Soc. Neurosci. 4, 473–490 10.1080/17470910802109939 18633842

[B7] Ben-Shakhar G. (1985). Standardization within individuals: a simple method to neutralize individual differences in skin conductance. Psychophysiology 22, 292–299 10.1111/j.1469-8986.1985.tb01603.x 4011799

[B8] Ben-Shakhar G. (2002). “A critical review of the control questions test (CQT),” in Handbook of Polygraph Testing, ed. Kleiner M. (San Diego: Academic Press), 103–126

[B9] Ben-Shakhar G. (2011). “Countermeasures,” in Memory Detection: Theory and Application of the Concealed Information Test, eds Verschuere B. Ben-Shakhar G. Meijer E. (Cambridge: Cambridge University Press), 200–214

[B10] Ben-Shakhar G. Dolev K. (1996). Psychophysiological detection through the guilty knowledge technique: effects of mental countermeasures. J. Appl. Psychol. 81, 273–281 10.1037/0021-9010.81.3.273 8690689

[B11] Ben-Shakhar G. Elaad E. (2002). Effects of questions’ repetition and variation on the efficiency of the guilty knowledge test: a reexamination. J. Appl. Psychol. 87, 972–977 10.1037/0021-9010.87.5.972 12395821

[B12] Ben-Shakhar G. Elaad E. (2003). The validity of psychophysiological detection of information with the guilty knowledge test: a meta-analytic review. J. Appl. Psychol. 88, 131–151 10.1037/0021-9010.88.1.131 12675401

[B13] Ben-Shakhar G. Meijer E. (2012). Skin conductance, respiration, heart rate, and P300 in the concealed information test: a meta analysis. Int. J. Psychophysiol. 85, 324 10.1016/j.ijpsycho.2012.06.095 24916920

[B14] Bradley M. T. Barefoot C. A. Arsenault A. M. (2011). “Leakage of information to innocent suspects,” in Memory Detection: Theory and Application of the Concealed Information Test, eds Verschuere B. Ben-Shakhar G. Meijer E. (Cambridge: Cambridge University Press), 187–199

[B15] Bradley M. T. Janisse M. P. (1981). Accuracy demonstrations, threat, and the detection of deception: cardiovascular, electrodermal, and pupillary measures. Psychophysiology 18, 307–315 10.1111/j.1469-8986.1981.tb03040.x 7291448

[B16] Carmel D. Dayan E. Naveh A. Raveh O. Ben-Shakhar G. (2003). Estimating the validity of the guilty knowledge test from simulated experiments: the external validity of mock crime studies. J. Exp. Psychol. Appl. 9, 261–269 10.1037/1076-898X.9.4.261 14664677

[B17] Coan J. A. Allen J. J. B. (2004). Frontal EEG asymmetry as a moderator and mediator of emotion. Biol. Psychol. 67, 7–49 10.1016/j.biopsycho.2004.03.002 15130524

[B18] Dawes R. M. (1979). The robust beauty of improper linear models in decision making. Am. Psychol. 34, 571–582 10.1037/0003-066X.34.7.571

[B19] Ekman P. (2001). Telling Lie. Clues to Deceit in the Marketplace, Politics, and Marriage. New York: Norton

[B20] Elaad E. (1990). Detection of guilty knowledge in real-life criminal investigations. J. Appl. Psychol. 75, 521–529 10.1037/0021-9010.75.5.521 1429348

[B21] Elaad E. Ben-Shakhar G. (2006). Finger pulse waveform length in the detection of concealed information. Int. J. Psychophysiol. 61, 226–234 10.1016/j.ijpsycho.2005.10.005 16712993

[B22] Elaad E. Ginton A. Jungman N. (1992). Detection measures in real-life criminal guilty knowledge tests. J. Appl. Psychol. 77, 757–767 10.1037/0021-9010.77.5.757 1429348

[B23] Farwell L. A. Donchin E. (1991). The truth will out: Interrogative polygraphy (“lie detection”) with event-related brain potentials. Psychophysiology 28, 531–547 10.1111/j.1469-8986.1991.tb01990.x 1758929

[B24] Fukuda K. (2001). Eye blinks: new indices for the detection of deception. Int. J. Psychophysiol. 40, 239–245 10.1016/S0167-8760(00)00192-6 11228351

[B25] Gamer M. (2011a). “Detecting concealed information using autonomic measures,” in Memory Detection: Theory and Application of the Concealed Information Test, eds Verschuere B. Ben-Shakhar G. Meijer E. (Cambridge: Cambridge University Press), 27–45

[B26] Gamer M. (2011b). “Detecting of deception and concealed information using neuroimaging techniques,” in Memory Detection: Theory and Application of the Concealed Information Test, eds Verschuere B. Ben-Shakhar G. Meijer E. (Cambridge: Cambridge University Press), 90–113

[B27] Gamer M. Berti S. (2010). Task relevance and recognition of concealed information have different influences on electrodermal activity and event-related brain potentials. Psychophysiology 47, 355–364 10.1111/j.1469-8986.2009.00933.x 20003148

[B28] Gamer M. Gödert H. W. Keth A. Rill H.-G. Vossel G. (2008a). Electrodermal and phasic heart rate responses in the guilty actions test: comparing guilty examinees to informed and uninformed innocents. Int. J. Psychophysiol. 69, 61–68 10.1016/j.ijpsycho.2008.03.001 18433904

[B29] Gamer M. Verschuere B. Crombez G. Vossel G. (2008b). Combining physiological measures in the detection of concealed information. Physiol. Behav. 95, 333–340 10.1016/j.physbeh.2008.06.011 18638496

[B30] Gamer M. Kosiol D. Vossel G. (2010). Strength of memory encoding affects physiological responses in the guilty actions test. Biol. Psychol. 83, 101–107 10.1016/j.biopsycho.2009.11.005 19931347

[B31] Gamer M. Rill H. G. Vossel G. Gödert H. W. (2006). Psychophysiological and vocal measures in the detection of guilty knowledge. Int. J. Psychophysiol. 60, 76–87 10.1016/j.ijpsycho.2005.05.006 16005091

[B32] Hira S. Furumitsu I. (2002). Polygraphic examinations in Japan: applications of the guilty knowledge test in forensic investigations. Int. J. Police Sci. Manag. 4, 16–27

[B33] Honts C. R. Devitt M. K. Winbush M. Kircher J. C. (1996). Mental and physical countermeasures reduce the accuracy of the concealed knowledge test. Psychophysiology 33, 84–92 10.1111/j.1469-8986.1996.tb02111.x 8570798

[B34] Kobayashi T. Yoshimoto K. Fujihara S. (2009). Jitsumu polygraph kensa no genjyo [The contemporary situation of field polygraph tests]. Jpn. J. Physiol. Psychol. Psychophysiol. 27, 5–15 10.5674/jjppp.27.5

[B35] Krapohl D. J. (2011). “Limitations of the concealed information test in criminal cases,” in Memory Detection: Theory and Application of the Concealed Information Test, eds Verschuere B. Ben-Shakhar G. Meijer E. (Cambridge: Cambridge University Press), 151–170

[B36] Littlewort G. Whitehill J. Wu T. Fasel I. Frank M. Movellan J. (2011). “The computer expression recognition toolbox (CERT),” in Proceedings of IEEE International Conference on Automatic Face and Gesture Recognition, 298–305

[B37] Lubow R. E. Fein O. (1996). Pupillary size in response to a visual guilty knowledge test: new technique for the detection of deception. J. Exp. Psychol. Appl. 2, 164–177 10.1037/1076-898X.2.2.164

[B38] Lykken D. T. (1959). The GSR in the detection of guilt. J. Appl. Psychol. 43, 385–388 10.1037/h0046060

[B39] Matsuda I. Hirota A. Ogawa T. Takasawa N. Shigemasu K. (2006). A new discrimination method for the concealed information test using pretest data and within-individual comparisons. Biol. Psychol. 73, 157–164 10.1016/j.biopsycho.2006.01.013 16504367

[B40] Matsuda I. Hirota A. Ogawa T. Takasawa N. Shigemasu K. (2009a). Within-individual discrimination on the concealed information test using dynamic mixture modeling. Psychophysiology 46, 439–449 10.1111/j.1469-8986.2008.00781.x 19170948

[B41] Matsuda I. Nittono H. Hirota A. Ogawa T. Takasawa N. (2009b). Event-related brain potentials during the standard autonomic-based concealed information test. Int. J. Psychophysiol. 74, 58–68 10.1016/j.ijpsycho.2009.07.004 19631702

[B42] Matsuda I. Nittono H. Ogawa T. (2011). Event-related potentials increase the discrimination performance of the autonomic-based concealed information test. Psychophysiology 48, 1701–1710 10.1111/j.1469-8986.2011.01266.x 21806637

[B63] Matsuda I. Nittono H. Ogawa T. (2012). Decomposing cognitive processes underlying the concealed information test by event-related potentials. Int. J. Biomed. Soft Comput. Hum. Sci. 18, 5–11

[B43] Matsuda I. Ogawa T. (2011). Improved method for calculating the respiratory line length in the concealed information test. Int. J. Psychophysiol. 81, 65–71 10.1016/j.ijpsycho.2011.06.002 21689693

[B44] Meijer E. H. Verschuere B. Ben-Shakhar G. (2011). “Practical guidelines for developing a CIT,” in Memory Detection: Theory and Application of the Concealed Information Test, eds Verschuere B. Ben-Shakhar G. Meijer E. (Cambridge: Cambridge University Press), 293–302

[B45] Nahari G. Ben-Shakhar G. (2011). Psychophysiological and behavioral measures for detecting concealed information: the role of memory for crime details. Psychophysiology 48, 733–744 10.1111/j.1469-8986.2010.01148.x 20958308

[B46] Nose I. Murai J. Taira M. (2009). Disclosing concealed information on the basis of cortical activations. Neuroimage 44, 1380–1386 10.1016/j.neuroimage.2008.11.002 19059486

[B47] Osugi A. (2010). Gap and connection between laboratory research and field application of the CIT in Japan. Int. J. Psychophysiol. 77, 238 10.1016/j.ijpsycho.2010.06.355

[B48] Osugi A. (2011). “Daily application of the concealed information test: Japan,” in Memory Detection: Theory and Application of the Concealed Information Test, eds Verschuere B. Ben-Shakhar G. Meijer E. (Cambridge: Cambridge University Press), 253–275

[B49] Pollina D. Dollins A. Senter S. Brown T. Pavlidis I. Levine J. (2006). Facial skin surface temperature changes during a “concealed information” test. Ann. Biomed. Eng. 34, 1182–1189 10.1007/s10439-006-9143-3 16786391

[B50] Reid J. E. (1947). A revised questioning technique in lie-detection tests. J. Crim. Law Criminol. 37, 542–547 20242527

[B51] Rosenfeld J. P. (2011). “P300 in detecting concealed information,” in Memory Detection: Theory and Application of the Concealed Information Test, eds Verschuere B. Ben-Shakhar G. Meijer E. (Cambridge: Cambridge University Press), 63–89

[B52] Rosenfeld J. P. Cantwell B. Nasman V. T. Wojdac V. Ivanov S. Mazzeri L. (1988). A modified, event-related potential-based guilty knowledge test. Int. J. Neurosci. 42, 157–161 10.3109/00207458808991608 3209369

[B53] Rosenfeld J. P. Labkovsky E. Winograd M. Lui M. A. Vandenboom C. Chedid E. (2008). The complex trial protocol (CTP): a new, countermeasure-resistant, accurate, P300-based method for detection of concealed information. Psychophysiology 45, 906–919 10.1111/j.1469-8986.2008.00708.x 18823418

[B54] Sartori G. Agosta S. Zogmaister C. Ferrara S. D. Castiello U. (2008). How to accurately detect autobiographical events. Psychol. Sci. 19, 772–780 10.1111/j.1467-9280.2008.02156.x 18816284

[B55] Sawada Y. Tanaka G. Yamakoshi K. (2001). Normalized pulse volume (NPV) derived photo-plethysmographically as a more valid measure of the finger vascular tone. Int. J. Psychophysiol. 41, 1–10 10.1016/S0167-8760(00)00162-8 11239692

[B56] Vandenbosch K. Verschuere B. Crombez G. De Clercq A. (2009). The validity of finger pulse line length for the detection of concealed information. Int. J. Psychophysiol. 71, 118–123 10.1016/j.ijpsycho.2008.07.015 18708102

[B57] Venables P. H. Mitchell D. A. (1996). The effects of age, sex and time of testing on skin conductance activity. Biol. Psychol. 43, 87–101 10.1016/0301-0511(96)05183-6 8805967

[B58] Verschuere B. Crombez G. Koster E. H. W. Van Bockstaele B. De Clercq A. (2007). Startling secrets: startle eye blink modulation by concealed crime information. Biol. Psychol. 76, 52–60 10.1016/j.biopsycho.2007.06.001 17656000

[B59] Verschuere B. De Houwer J. (2011). “Detecting concealed information in less than a second: response latency-based measures,” in Memory Detection: Theory and Application of the Concealed Information Test, eds Verschuere B. Ben-Shakhar G. Meijer E. (Cambridge: Cambridge University Press), 46–62

[B60] Verschuere B. Meijer E. De Clercq A. (2011). Concealed information under stress: a test of the orienting theory in real-life police interrogations. Legal Criminol. Psychol. 16, 348–356

[B61] Winograd M. R. Rosenfeld J. P. (2011). Mock crime application of the complex trial protocol (CTP) P300-based concealed information test. Psychophysiology 48, 155–161 10.1111/j.1469-8986.2010.01054.x 20557482

[B62] Zhao M. Zheng C. Zhao C. (2011). A new approach for concealed information identification based on ERP assessment. J. Med. Syst. 36, 2401–2409 10.1007/s10916-011-9707-0 21499695

